# Hepatitis C virus transmission cluster among injection drug users in Pakistan

**DOI:** 10.1371/journal.pone.0270910

**Published:** 2022-07-15

**Authors:** Kashif Iqbal Sahibzada, Lilia Ganova-Raeva, Zoya Dimitrova, Sumathi Ramachandran, Yulin Lin, Garrett Longmire, Leonard Arthur, Guo-liang Xia, Yury Khudyakov, Idrees Khan, Saima Sadaf

**Affiliations:** 1 School of Biochemistry and Biotechnology, University of the Punjab, Lahore, Pakistan; 2 Division of Viral Hepatitis, Center of Disease Control and Prevention, Molecular Epidemiology and Bioinformatics, Atlanta, GA, United States of America; 3 University of Peshawar, Peshawar, KPK, Pakistan; Centre de Recherche en Cancerologie de Lyon, FRANCE

## Abstract

Hepatitis C virus (HCV) infections are public health problem across the globe, particularly in developing countries. Pakistan has the second highest prevalence of HCV infection worldwide. Limited data exist from Pakistan about persons who inject drugs (PWID) and are at significant risk of exposure to HCV infection and transmission. Serum specimens (n = 110) collected from PWID residing in four provinces were tested for molecular markers of HCV infection. Next generation sequencing (NGS) of the hypervariable region (HVR1) of HCV and Global Hepatitis Outbreak and Surveillance Technology (GHOST) were used to determine HCV genotype, genetic heterogeneity, and construct transmission networks. Among tested specimens, 47.3% were found anti-HCV positive and 34.6% were HCV RNA-positive and belonged to four genotypes, with 3a most prevalent followed by 1a, 1b and 4a. Variants sampled from five cases formed phylogenetic cluster and a transmission network. One case harbored infection with two different genotypes. High prevalence of infections and presence of various genotypes indicate frequent introduction and transmission of HCV among PWID in Pakistan. Identification of a transmission cluster across three provinces, involving 20% of all cases, suggests the existence of a countrywide transmission network among PWIDs. Understanding the structure of this network should assist in devising effective public health strategies to eliminate HCV infection in Pakistan.

## Introduction

Chronic hepatitis C virus (HCV) infection may lead to cirrhosis and hepatocellular carcinoma. An estimated 71 million people are infected with HCV globally [1, 2]. HCV infection is endemic in Pakistan [3], with an seroprevalence as high as 14.64% in Punjab [4], estimated 6.1% active HCV infections [5] and approximately 10 million people infected [6]. The main transmission routes for HCV include contaminated surgical tools and syringes, transfusion of contaminated blood or blood products [7], and drug abuse [8]. People who inject drugs (PWID) [9, 10], people infected with HIV [10], people with the history of incarceration, and men who have sex with men (MSM) account for high-risk of HCV infection and transmission [11]. Comprehensive assessment of HCV infected PWID [8] is essential for implementation of effective healthcare prevention and HCV control strategies [12, 13]. A recent study estimated that ~ 7.6 million Pakistanis experienced drug addiction [14], and this number increases by ~4,000 per year [15]. Recent estimates show that approximately half of PWIDs in the Middle East are infected with HCV with the largest numbers found in Pakistan [14]. Meta-analysis of HCV prevalence in Pakistan demonstrates that even though the pooled mean prevalence in the general population is approx. 6.2%, it is 53.6% among Pakistani PWIDs [6]. According to the UN Office of Drugs and Crime there are 6.7 million drug users in Pakistan, making PWIDs a major contributor to the HCV epidemic in the country. Low screening rates of blood donations and paid donations by PWIDs, contribute to the population’s exposure to HCV [7].

HCV is a single-stranded (+) RNA virus. HCV genome translates into a single polyprotein, which is processed into 3 structural (core, E1 and E2) and 7 non-structural proteins (p7, NS2, NS3, NS4A, NS4B, NS5A, and NS5B) [16]. HCV is highly prone to mutations, and exists as a population of closely related intra-host variants in each infected person [16]. Genetic heterogeneity is not uniformly distributed along the HCV genome, with hypervariable region 1 (HVR1) being located at the N-terminus of E2 [17, 18]. Extensive heterogeneity of HVR1 is used for identification of transmission networks [19], for detection of HCV transmission during outbreak investigation [10, 20], and estimation of HCV evolution [18]. HCV had been classified into 7 genotypes and 67 subtypes [21], with a novel genotype 8 having been recently identified in India [22]. Prevalence of various genotypes is not uniform in different countries. Genotype 3a is most prevalent in Pakistan, accounting for 63% of all HCV infections [3].

Next Generation Sequencing (NGS) allows for in-depth characterization of intra-host viral populations [19, 23]. NGS of HCV HVR1 produces sequence reads from multiple HVR1 variants [24]. The CDC-developed novel technology called the Global Hepatitis Outbreak and Surveillance Technology (GHOST) [25] processes these HVR1 reads into haplotypes, assesses their frequency, and detects and visualizes transmission clusters and networks [24]. Here, we used GHOST to characterize HCV strains and identify transmission linkages among PWID in Pakistan.

## Materials and methods

### Specimens

Blood samples (n = 110) were collected during 2019–2020 in Pakistan from PWID. Written informed consent was obtained from the participants along with a questionnaire about gender, age, risk behavior, marital status and frequency of injecting drugs. The ethics committee at the School of Biochemistry and Biotechnology, University of the Punjab approved the research.

### Testing for HCV infection

All specimens were tested for anti- HCV antibody with VIDAS Anti-HCV Assay (bioMerieux, France). Anti-HCV positive samples (n = 52) were further tested for HCV RNA using GeneXpert HCV viral load test (Cepheid, Inc., California, USA). The HCV RNA positive specimens (n = 38) were genotyped using HCVg DirectTest (GenMark Diagnostics, Inc., California, USA).

### Nucleic acid extraction and amplification of HVR1 region

Anti-HCV positive and HCV RNA positive samples (n = 38) were used for HCV molecular analysis. Total nucleic acid was extracted from 200ul serum using MagNA Pure LC instrument (Roche Life Science), and Total Nucleic Acid (TNA) isolation kit (Roche Diagnostics, Mannheim, Germany). Isolated TNA was reverse transcribed by One-step PCR (Qiagen, Inc., Germany) using gene specific primers targeted to the fragment of the E gene (nt 1325–1619) which contains the HVR1 region [10]. A nested product was then PCR amplified with barcoded gene specific PCR primers (1360F /1610R). Products were visualized by gel electrophoresis system. The HVR1 amplicons were also verified by Sanger consensus sequencing using Big Dye v3.1 chemistry and Applied Biosystems 3130xl Genetic Analyzer (ThermoFisher). The consensus contigs were assembled and analyzed by SeqManPro software (DNASTAR, Wisconsin) and confirmed with the NCBI genotyping tool https://www.ncbi.nlm.nih.gov/projects/genotyping/formpage.cgi.

### Next generation sequencing (NGS)

The nested barcode HCV HVR1 positive PCR products were cleaned using AMPure XP beads (Beckman-Coulter), followed by an Index PCR using indexes and adapters, as required for NGS and demultiplexing. The products of the index PCR were purified using AMPure XP beads and quantified on Tape station instrument (Agilent, California, USA) according to manufacturer’s instructions. Normalization was done by taking the appropriate volume of each fragment and mixing together to create an equimolar pooled library of all positive specimens (n = 25). The pooled library size was checked after purification on 4150 Tape station system (Agilent Inc., USA) followed by the dilution of library to 3nmol/l for the MiSeq sequencing procedure using v3 chemistry on Illumina MiSeq instrument and sequenced on MiSeq Instrument (Illumina Inc, USA).

### GHOST analysis

Processing of the paired reads, demultiplexed by index as obtained from the Illumina runs, was done by GHOST. The sequences were first passed through the quality control (QC) module which includes data filtering steps to merge the paired reads and determine their unique haplotype frequencies and genotypes. Sequence haplotypes that have passed the QC processing were further used in GHOST to analyze HCV heterogeneity and presence of transmission links using all the haplotypes with frequency 10 or more. The analysis module uses hamming distance between those haplotypes by comparing the population of each pair of cases. Two cases were linked by transmission, if the distance between them is calculated less than the threshold value of 0.037 [24].

To visualize the quasispecies network and inter-host haplotype sharing between the cases where transmission was detected, we used k-step networks. The links between the nodes of the networks belong to the union of all minimum spanning trees that is calculated using the Hamming distances between the haplotypes [26]. The k-step networks built by GHOST were derived from all the NGS unique haplotypes found above a predefined frequency [25].

Multiple sequence alignments and phylogenetic trees were created in MEGAv10.1.8.

## Results

### Sampled population

Serum specimens (n = 110) were collected mainly from Punjab and KPK, with only 5.4% being sampled from Baluchistan and Sindh (S1 Table). The median age of the participants was 27 years, with 42% being 21–30 years old (S1A Fig). Most were using heroin, followed by opiates and cocaine (S1B Fig). Of the 110 samples, 52 (47.2%) were anti-HCV positive, of which 38 (73.1%) were HCV RNA positive. Sharing of needles was associated with HCV past or current infection, (*P* < .001) (S1 Table). The number of males significantly exceeded the number of females (S1 Table) in this study. Among 11 females, 6 were anti-HCV positive and none were HCV RNA positive.

### HCV phylogenetic analysis

**Fig 1:**
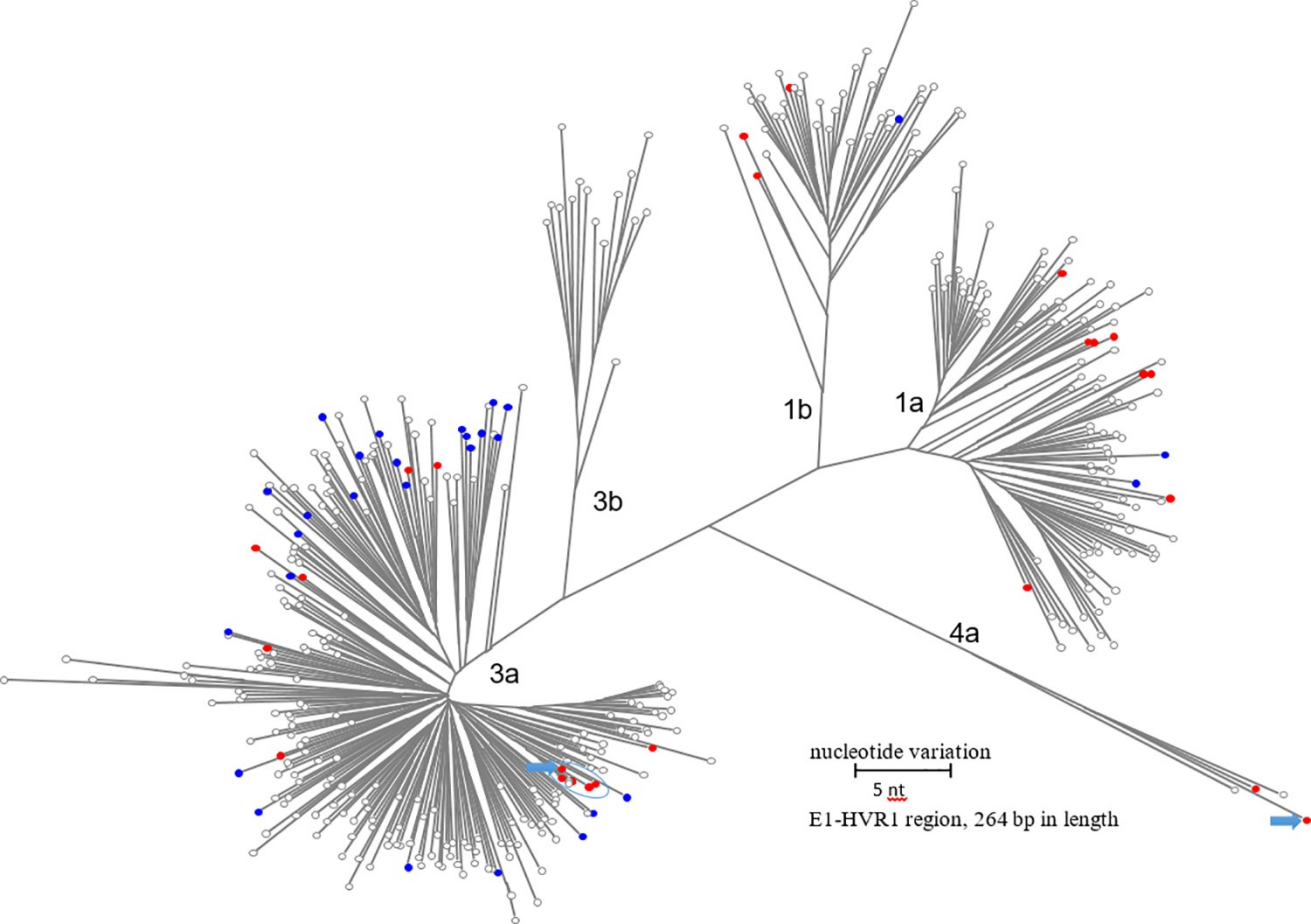
**Unrooted maximum likelihood phylogenetic tree of HVR1 sequences 264 bp of length (NGS major haplotypes only) identified in this study (red) and shown in the context of GenBank reference sequences (grey) and other HVR1 sequences from Pakistan (blue).** Clustered sequences are encircled. Arrows indicate HVR1 sequences of genotype 3a and 4a derived from PK-3 (mixed genotype infection).

HCV HVR1 sequences were obtained from 25 HCV RNA positive samples. Phylogenetic analysis allowed to classify the HCV strains into genotypes 1a, 1b, 3a, and 4a (S1C Fig). Genotype 3a was found to be most prevalent, followed by genotype 1a, 1b and 4a. HCV genotype 4a was detected in Pakistan for the first time. Fig 1 shows a phylogenetic tree of major haplotypes identified in each sample and relevant GenBank sequences. The HVR1 sequences identified were scattered across the tree and intermixed with sequences from GenBank. However, five HVR1 sequences formed a tight cluster within the genotype 3a branch.

### HCV transmission cluster analysis

**Fig 2:**
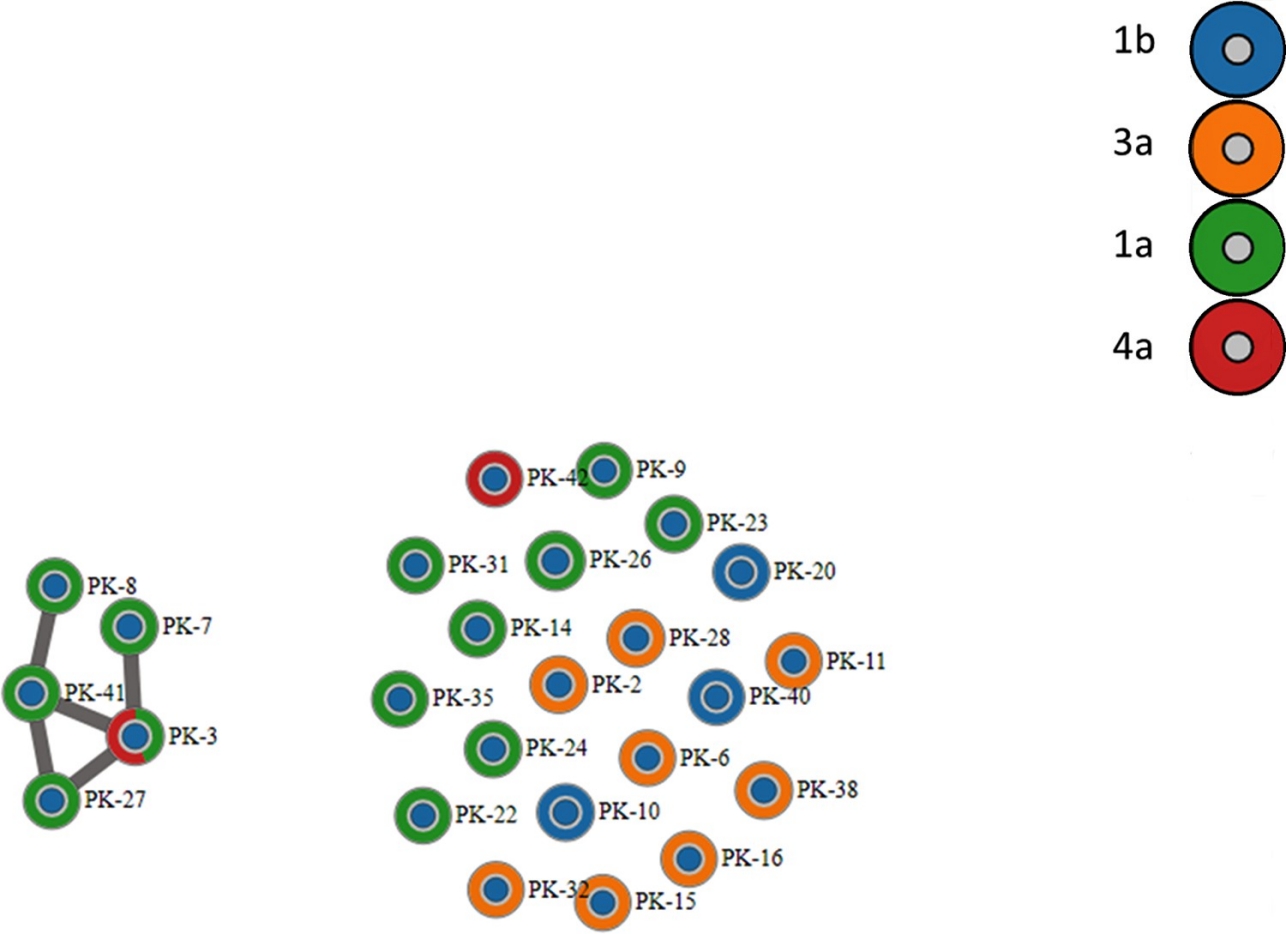
The GHOST HCV transmission network. Each node represents a person. Link is drawn if the minimal hamming distance between sequences from two samples is smaller than the established genetic relatedness threshold [20]. Genotypes are color-coded. Color of the node core identifies members of the transmission cluster (yellow) and unlinked cases (blue).

Intra-host HCV HVR1 populations from the 25 samples were tested by GHOST to confirm close genetic relatedness of the five HCV variants in the genotype 3a phylogenetic cluster. As shown in Fig 2, GHOST identified a HCV transmission cluster involving these five cases (20% of all sequenced samples). In the GHOST transmission network, each node represents a person and the line between two persons indicates a transmission link. Transmission link is defined by minimal genetic distance below 0.037 between intra-host HCV HVR1 populations from two cases [24]. All five cases in this transmission cluster were males who reported daily use of drugs and needle sharing. These cases were sampled from different geographic locations. PK-3, PK-27 and PK-41 were from Punjab, whereas PK-7 and PK-8, who have only one link in the transmission network, were from Sindh and KPK, correspondingly. The cases also differ in reported drug use; three reported use of heroin and two reported cocaine.

### Transmission cluster k-step network

**Fig 3:**
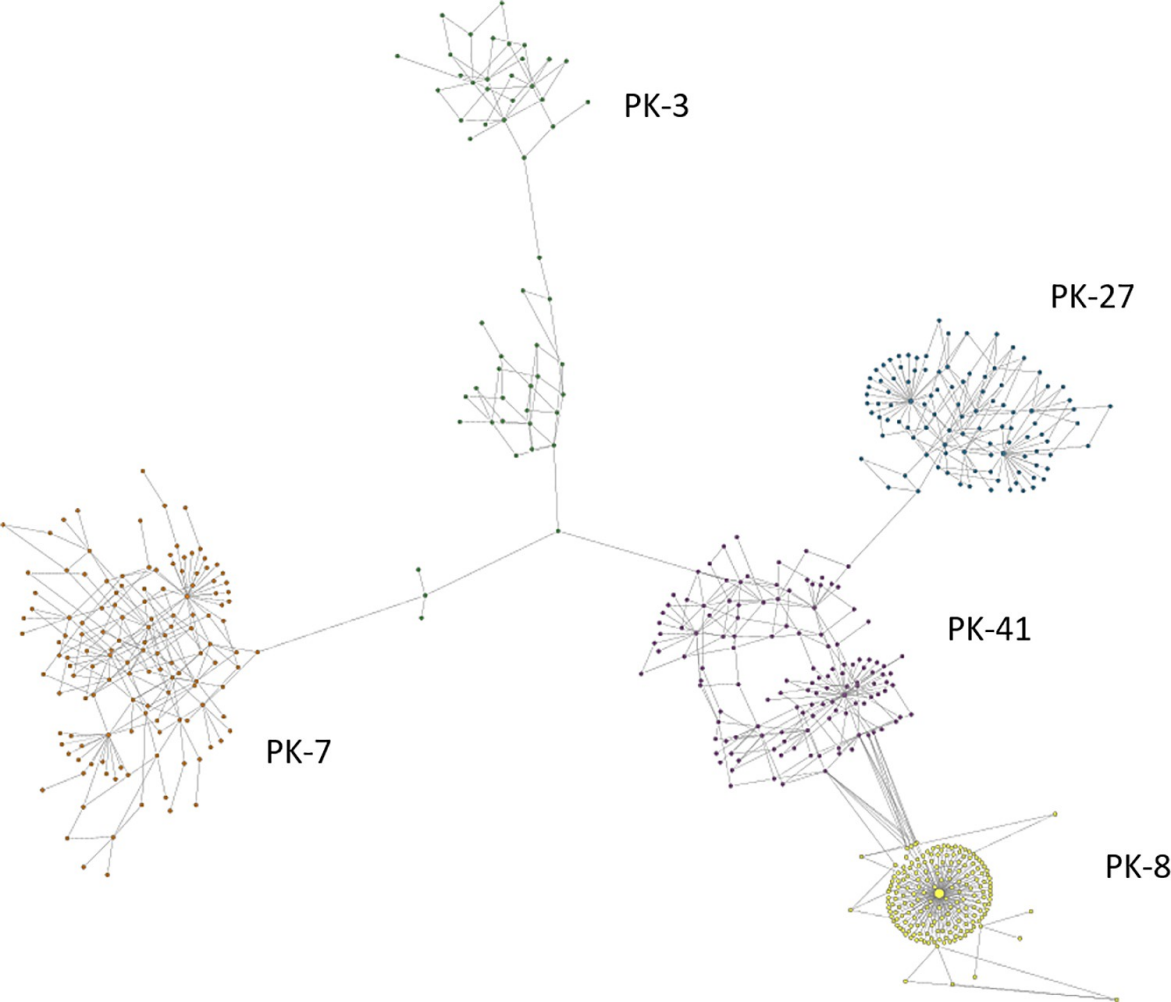
K-step network of the transmission cluster. Color identifies haplotypes from each case. Only genotype 3a haplotypes from PK-3 are shown.

**Fig 4:**
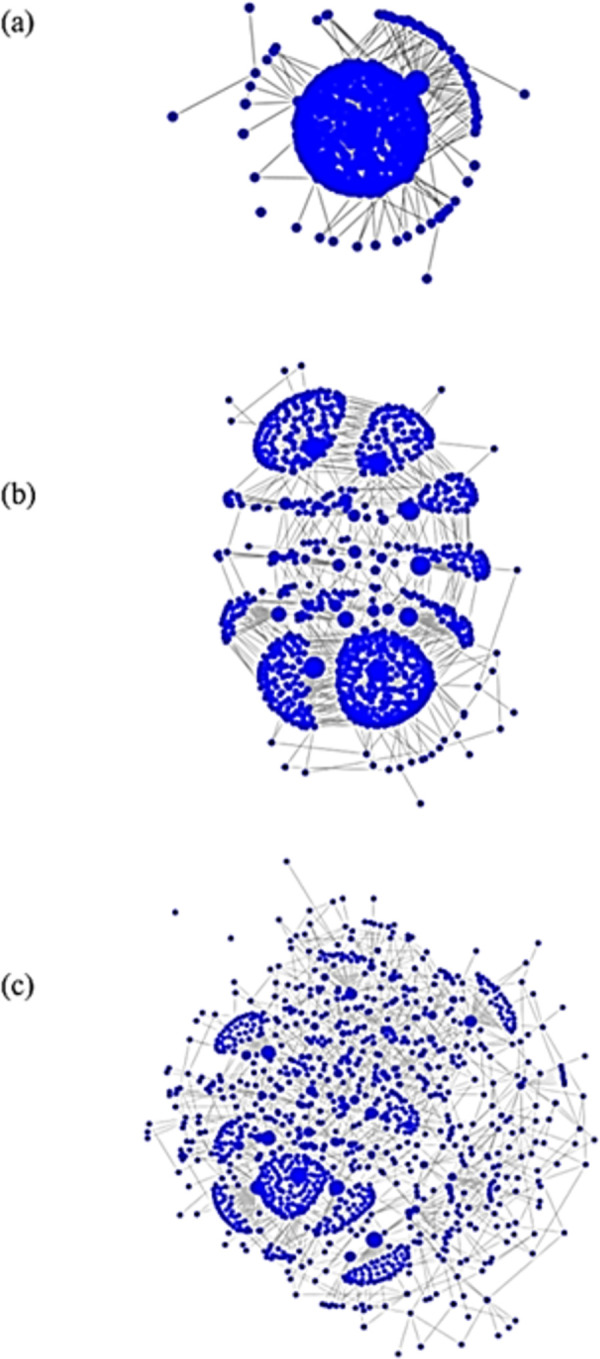
K-step networks of HCV HVR1 sequences from 3 samples. Each node represents a unique haplotype, the diameter of the node is proportional to the haplotype frequency and links between adjacent nodes belong to the union of all minimum spanning trees (a) Low diversity intra-host HCV HVR1 population from PK-9 (0.00512), genotype 3a; (b) average diversity population from PK-20 (0.0142), genotype 1b; (c) high diversity population from PK-23 (0.0226), genotype 3a.

Intra-host genetic diversity and relatedness among the five HCV cases in the transmission cluster were visualized using k-step network constructed from the genotype 3a sequences sampled from these cases (Fig 3). The intra-host HCV HVR1 populations showed a broad range of heterogeneity (Table 1). In the k-step network, the structure of intra-host HCV HVR1 population from PK-8 is star-like and the least heterogeneous with nucleotide diversity of 0.00654, whereas population structures from the other cases in the cluster are rather dispersed, with nucleotide sequence diversity ranging between 0.01429–0.02883. This diversity and structure of intra-host populations was within the range found among the linked cases (Table 2, Fig 4).

**Table 1:** Intra-host HCV HVR1 heterogeneity.

Per specimen	No. haplotypes	No. reads	Total diversity	Frequency of Major
Average	4850	15259	0.015462	4059
Min	1443	5019	0.005123	445
Max	9926	19329	0.047517	9604

**Table 2:** Properties of members of the transmission cluster.

Case	Gender	Drug used	Age	Needle sharing	Frequency of drug use	Province	Total genetic diversity
3	Male	Cocaine	22	Yes	Daily	Punjab	0.02883
7	Male	Heroine	17	Yes	Daily	Sindh	0.02139
8	Male	Cocaine	23	Yes	Daily	KPK	0.00654
27	Male	Heroine	27	Yes	Daily	Punjab	0.01429
41	Male	Heroine	45	Yes	Daily	Punjab	0.02215

### Mixed infection

**Fig 5:**
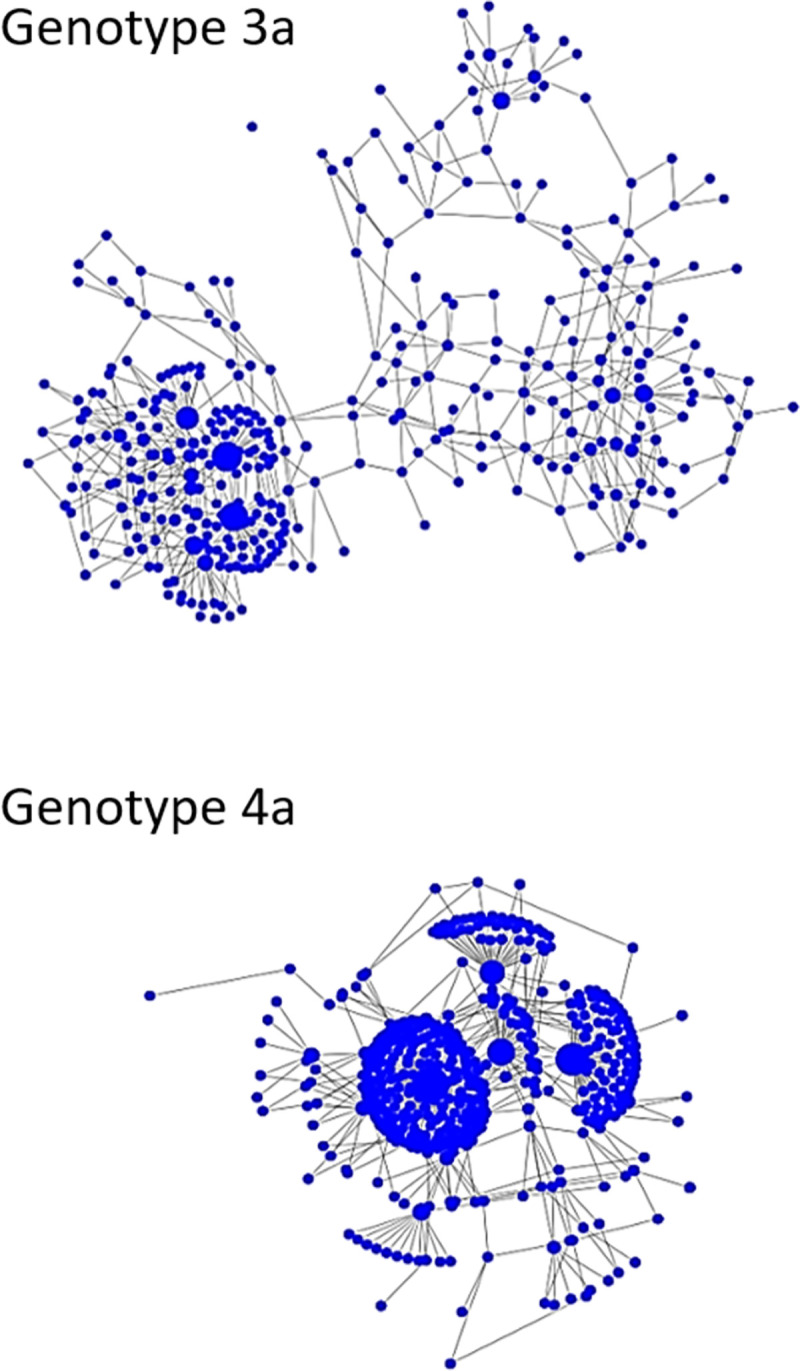
PK-3 mixed infection with two HCV genotypes.

NGS sequencing and haplotype genotyping revealed the presence of a mixed genotype infection in PK-3. Repeated NGS sequencing confirmed the presence of genotypes 3a (44% of the reads) and 4a (56% of reads). PK-3 was using heroin and participated in daily group injections with sharing needles. The k-step network of the PK-3 HCV HVR1 haplotypes is shown in Fig 5. The 3a haplotypes (48% of the total) have a greater genetic diversity (0.0288) compared to the 4a haplotypes (0.0128). Frequency of the major genotype 3a and 4a haplotypes was 470 and 1814, correspondingly. The 4a haplotypes from PK-3 and PK-42 are genetically distant (Fig 1).

## Discussion

Injection drug use was shown to be associated with high prevalence of HCV infection [27] and with multiple introductions of different HCV strains into PWID communities [10, 20]. Indeed, we found that 42.3% of samples collected from PWID were anti-HCV positive and 34.6% were HCV RNA positive. Taken together with identification of 4 genotypes and many HCV strains, these data indicate a frequent exposure to HCV infections and effective HCV transmission among PWID in Pakistan. Dominance of HCV genotype 3a, presence of genotype 1a and rare presence of genotype 1b in Pakistan have been reported [28]. Intermixing of the HCV variants from PWID tested here and other reference HCV variants from Pakistan in the phylogenetic tree indicates circulation of genetically similar HCV strains among different populations in the country. However, to our knowledge, presence of HCV genotype 4a strains in Pakistan is reported for the first time and adds validity to the hypothesis that PWID are exposed to a great variety of HCV strains.

Identification of a phylogenetic cluster of five closely related HCV strains of genotype 3a indicates that some HCV strains from PWID are more genetically linked than others. Such close genetic linkage among HCV strains may result from frequent HCV transmission. GHOST testing confirmed that genetic distances among intra-host HCV HVR1 populations from these five cases in the cluster are consistent with linkage by transmission among these cases. The transmission network generated by GHOST shows that three cases, PK-3, PK-27 and PK-41, form a clique, whereas PK-7 is linked only to PK-3 and PK-8 to PK-41. Analysis of heterogeneity of intra-host HCV HVR1 populations showed that these linkages cannot be an artifact of high diversity of these populations, with PK-8 having low diversity and the other four cases having diversity close to the average for unlinked cases.

The cases from the transmission cluster reside in 3 provinces. Three cases forming the clique are from Punjab and the other two cases with a single link are from KPK and Sindh. This finding suggests the existence of a large country-wide transmission network among PWID. There are additional observations, which by themselves do not provide proof but are consistent with this supposition: (a) 20% of cases from a small sample of HCV HVR1 positive cases (n = 25) found in the transmission cluster; (b) lack of sharing of HCV HVR1 haplotypes between cases linked by transmission in the GHOST network; (c) members of the cluster reporting use of two drugs, heroine (n = 3) and cocaine (n = 2); and (d) a broad age range (17–45 years) among the cluster members. Indeed, large high-risk populations can be expected to have a greater capacity than small populations to support continuous circulation of closely related HCV strains at the level sufficient for the detection of cases infected with these strains from a limited, random sample, and to involve people with a large difference in age who report exclusive or preferential use of two different drugs. Detection of a transmission cluster from a small high-risk population would be rather indicative of a recent outbreak. Recency of infection and direct transmission usually observed during outbreaks result in frequent sharing of at least some HCV HVR1 haplotypes of intra-host viral populations among outbreak cases [29]. However, as can be seen in the transmission k-step network, no such sharing was observed, which can be explained by sampling from a large high-risk population with well-established HCV infections. Lack of haplotype sharing also means that links shown among the cluster members sampled at random most likely do not represent direct transmission but indicate membership in a large transmission network and mark close genetic relationships among the members.

Sharing needles and syringes increases the risk of HCV [30, 31]. In Pakistan, sharing needles and syringe reuse have been reported as the major cause of HCV transmission [32]. We observed here that all PWID who reported sharing needles (n = 30) or regularly injected in groups (n = 23) had current HCV infection. All five cases in the transmission cluster reported daily use of drugs and needle sharing, indicating a high risk of exposure to HCV.

PK-3 from the cluster is infected with two HCV strains from genotypes 3a and 4a. Mixed-genotype infections can be frequently found in communities with high-risk of HCV exposure such as PWID [10, 20, 23, 28]. Members of PWID community with mixed-genotype HCV infections usually occupy central positions in transmission networks [33]. Probability of mixed infections is expected to increase in large high-risk communities, which may experience frequent introduction and persistent circulation of various HCV strains, thus establishing favorable conditions for exposure to more than one HCV strain and resulting in co- or super-infections [33]. Herein, this observation is consistent with the supposition that PK-3, as part of the transmission cluster, belongs to a large PWID transmission network.

Although all observations presented here are coherent, it is important to note that inferences from genetic analyses of intra-host HCV HVR1 variants from 25 specimens might be affected by sampling biases, thus potentially limiting generalizability of the findings. Nevertheless, considering the unique nature of these findings, these data warrant further investigation into the existence of a countrywide transmission network among PWID.

The Welcome Trust has estimated that further improvements in blood safety and infection control, expansion or creation of PWID harm reduction services, and extensive screening for HCV with concomitant offer of DAAs all are necessary to reduce the burden of HCV, especially in China, India, and Pakistan [12]. The top suggested approaches to curbing the HCV epidemic in Pakistan and achieving the 2030 WHO HCV elimination goals include targeting for treatment persons with cirrhosis and PWIDs [13].

In conclusion, the data presented here indicate introduction of numerous HCV strains to PWID in Pakistan and as the clustered cases are from different locations, suggest the existence of a large countrywide HCV transmission network. Further characterization of this network is important for understanding HCV transmission among PWID and for devising effective public health interventions to eliminate HCV infection in Pakistan.

Disclaimers: The findings and conclusions in this report do not necessarily reflect the official position of the Centers for Disease Control and Prevention, or the authors’ affiliated institutions.

Use of trade names and commercial sources is for identification only and does not imply endorsement by the Centers for Disease Control and Prevention, the Public Health Service, or the US Department of Health and Human Services.

## Supporting information

S1 Fig (a) Age distribution among PWID; (b) Drug use distribution; and (c) Genotype distribution.
(TIF)

S1 Table HCV infection status and demographic and injection drug use parameters. (DOCX)
