# Supplementary figures and images for: Hepatitis C virus transmission cluster among injection drug users in Pakistan
Source: PLoS One. 2022 Jul 15;17(7):e0270910. doi: 10.1371/journal.pone.0270910 (PMC9286280; doi:10.1371/journal.pone.0270910)

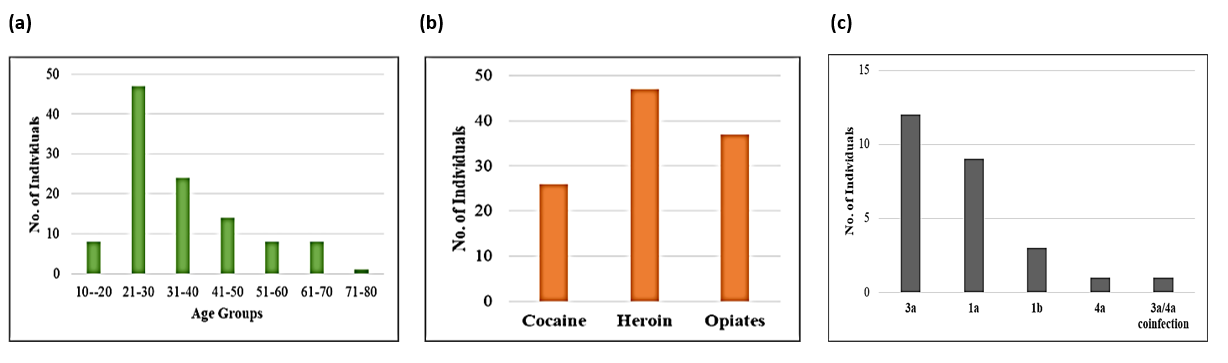

Supplement: S1 Fig — (a) Age distribution among PWID; (b) Drug use distribution; and (c) Genotype distribution. (TIF) [file pone.0270910.s001.tif]
