# Supplementary material for: Hepatitis C virus transmission cluster among injection drug users in Pakistan
Source: PLoS One. 2022 Jul 15;17(7):e0270910. doi: 10.1371/journal.pone.0270910 (PMC9286280; doi:10.1371/journal.pone.0270910)
Supplement: S1 Table — (DOCX) [file pone.0270910.s002.docx]

|  | | HCV RNA detection (NAT) | | | Chi-square | p-value |
| --- | --- | --- | --- | --- | --- | --- |
|  |  | NAT  Positive | NAT  Negative | Total (%) |  |  |
| GENDER | Male | 34 | 56 | 90 (81.82) | 2.287 | .13 |
|  | Female | 4 | 16 | 20 (18.18) |  |  |
| Category | Cocaine | 7 | 19 | 26 (23.64) | 7.706 | .02 |
|  | Heroin | 23 | 24 | 47 (42.73) |  |  |
|  | Opiates | 8 | 29 | 37 (33.64) |  |  |
| Group Injecting | Never | 3 | 64 | 67 (60.91) | 76.098 | < .001 |
|  | Sometimes | 12 | 8 | 20 (18.18) |  |  |
|  | Always | 23 | 0 | 23 (20.91) |  |  |
| Frequency | Daily | 28 | 30 | 58 (52.72) | 76.905 | < .001 |
|  | Weekly | 9 | 23 | 32 (29.00) |  |  |
|  | Monthly/Occasionally | 1 | 19 | 20 (18.18) |  |  |
| Sharing Needles | Yes | 30 | 0 | 30 (27.27) | 78.158 | < .001 |
|  | No | 8 | 72 | 80 (72.73) |  |  |
